# Signe de Mont Fuji en postopératoire d’un épendymome du V3: cas clinique et revue de la literature

**DOI:** 10.11604/pamj.2016.24.191.9265

**Published:** 2016-07-01

**Authors:** Abderrahmane Boumadiane, Ali Derkaoui, Abdelkarim Shimi, Mohamed Khatouf

**Affiliations:** 1 Service de Réanimation Polyvalente A1 CHU Hassan II, Fès, Maroc

**Keywords:** Signe de «Mont Fuji», pneumocéphalie compressive, complication post-opératoire, Mount Fuji sign, compressive pneumocephalus, ependymoma, post-operative complication

## Abstract

Le signe de « Mont Fuji » appelé autrement une pneumocéphalie compressive constitue une complication post-operatoire redoutable en neurochirurgie. Nous rapportons le cas clinique d'un patient de 10 mois, hospitalisé en réanimation pour prise en charge post-operatoire d'une chirurgie pour épendymome du troisième ventricule, dont l'evolution a été marquait par la survenue d'une pneumocéphalie compressive post-operatoire précoce, responsable d'une aggravation neurologique et hemodynamique. A travers cette observation, on met en évidence la possibilité de survenue d'un tel événement indésirable, Ainsi que des moyens thérapeutiques et surtout préventifs de cette complication.

## Abstract (en)

Mount Fuji sign also known as compressive pneumocephalus is a redoubtable postoperative neurosurgical complication. We report the clinical case of a 10 months-old patient, hospitalized in reanimation for postoperative management following surgery for ependymoma of the third ventricle. The evolution was marked by the occurrence of early postoperative compressive pneumocephalus, responsible for neurological and hemodynamic worsening. This study highlights the possibility of occurrence of such an adverse event, as well as the therapeutic and especially preventive measures for this complication.

## Introduction

La pneumocéphalie se définie par la présence d´air en intracrânien, c´est une complication possible en postopératoire d´une resection chirurgicale de l´épendymome du troisième ventricule, cette complication peut rester asymptomatique et spontanément résorbable. Néanmoins elle peut être compressive et responsable d´un syndrome d´hypertension intracrânienne (HTIC). Le but de notre travail est de décrire cette pathologie grave, afin d´instaurer des moyens de prévention.

## Patient et observation

**Figure 1:**
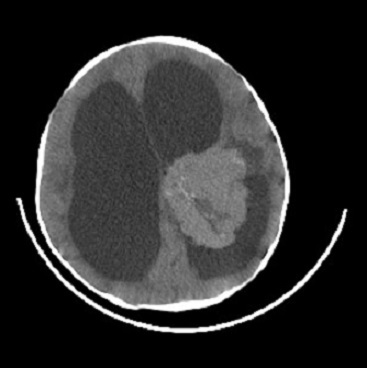
Image scannographique du patient objectivant un épendymome du V3 avec hydrocephalie quadriventriculaire

**Figure 2:**
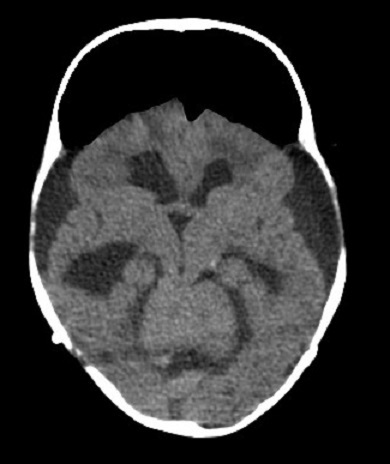
Image scanographique postopératoire du meme patient objectivant: effacement des sillons corticaux, écrasement des ventricules cérébraux, compression des lobes frontaux réalisant le classique signe du « mont Fuji »

Nous rapportons l'observation du nourisson de 10 mois, suivi en service de neurochirurgie pour HTIC en rapport avec épendymome du troisième ventricule (V3) objectivé sur le scanner cerebrale (Figure 1). Le patient a bénéficié d´une resection neurochirurgicale de la tumeur puis fut hospitalisé au service de Réanimation Polyvalente pour prise en charge post-opératoire: l´intervention chirurgicale a été réalisée sous anesthèsie générale, la chirurgie a consisté à une craniotomie avec resection totale de la tumeur. L´evolution post-operatoire a été marquait par un syndrome d´HTIC et d´une deterioration neurologique et hemodynamique, le scanner cérébrale de contrôle (Figure 2) a objectivé; un effacement des sillons corticaux, un écrasement des ventricules cérébraux, une compression des lobes frontaux réalisant le classique signe du « mont Fuji ». La conduite à tenir a consisté en une hospitalisation en réanimation, approfendissement de la sedation, hyperventilation. L'évolution était fatale, marquée par une aggravation neurologique et hemodynamique rapide et refractaire, la pneumocéphalie était indirectement impliquée dans le décès de ce patient.

## Discussion

La pneumocéphalie postopératoire est souvent observée chez les patients neurochirurgicaux au cours de la période postopératoire précoce. La distinction d´une pneumocéphalie simple asymptomatique de la pneumocéphalie compressive (Mont Fuji) est d´une importance évidente car cette dernière met en jeu le pronostic vital des patients [1]. Reasonner et al. [2] ont décrit que 66% des scanners post-craniotomie objectivent 5 à 10% de volume intracrânien occupé de pneumocéphalie. La chirurgie des tumeurs de la fosse cérébrale postérieure est aussi pourvoyeuse de cette complication post-opératoire. La pneumocéphalie compressive est une urgence neurochirurgicale rare mais traitable. Un diagnostic rapide et précis de pneumocéphalie de tension exige un indice élevé de suspicion clinique corroboré par l´imagerie [3]. La signification scanographique du caractère compressif de la pneumocéphalie est identifiée par le signe de « Mont Fuji » [4] qui est équivalent de l´hypertension de l´air sous-dural comprimant et séparant les lobes frontaux. Aussi par le signe de « bulles d´air »: caractérisé par la présence de multiple bulles d´air disséminées autour des citernes cérébrales en postopératoire d´une neurochirurgie [5].

Les principaux facteurs de risque pourvoyeur de la pneumocéphalie compressive sont variés: Traumatisme crânien, craniotomie, chirurgie pour tumeur des sinus paranasaux, trou de trépan en chirurgie de l´HSDC sont quelques-unes des causes reconnues. En anesthésie, le protoxyde d´azote comme agent anesthésique a été impliqué initialement dans la genèse de la pneumocéphalie avant d être innocenté par nombre d´études [5]. L´utilisation de l´oxygénation hyperbare et le mode de ventilation à pression positive continue (CPAP) sont également impliqués dans la pneumocéphalie [6]. Le mécanisme de la pneumocéphalie compressive trouve ces explications dans deux théories; la première dite « théorie de la soupape a billes »: qui s´exprime par la pénétration de l´air en intra crânien à travers le défect osseux (lors de la craniotomie) au moment où la pression externe dépasse la pression intracrânienne, la deuxième est la « théorie de la bouteille inversée »: qui s´explique par l´entrée de l´air en intracrânien pour compenser la négativation de la pression intracrânienne à l´occasion d´un drainage de liquide cérébro-spinal [1, 7, 8].

Les mesures préventives sont essentielles et concernent principalement la position peropératoire de la tête, et les moyens de réexpansion cérébrale peropératoire [1, 7]; à fin de faciliter la sortie de l´air sous-durale, il est important de souligner l´intérêt de repositionner la tête de façon à ce que l´orifice la craniotomie soit situé au point le plus culminant de la cavité crânienne au moment de la fermeture de la dure-mère [1], aucune étude n´a prouver l´impact de la position de la tête postopératoire sur la survenue de la pneumocéphalie. Le système de drainage postopératoire reste préférable qui il soit clos et non aspiratif [8], le volet médical préventif a pour objectif d´éviter la compression cérébrale et d´assurer une bonne hydratation qui permet de maintenir le volume cérébral [1]. Il est important de rappeler que dans notre cas étudié, on n´a pas utilisé le protoxyde d´azote du point de vue anesthésique, qui a été initialement incriminé avant qui il a été récusé par plusieurs études dans la survenue de la pneumocéphalie postopératoire [5]. Les principales moyens thérapeutiques à instaurer dans la prise en charge de la pneumocéphalie postopératoire incluent l´aspiration de l´air intracrânien, l´oxygénothérapie efficace et maintien d´une hydratation optimale pour favoriser la résorption de pneumocéphalie[9, 10]. La reprise chirurgicale reste exceptionnelle [10] mais la décision de recours à la chirurgie décompressive doit être prise devant l´importance de volume de la pneumocéphalie qui devient alors compressive et provoque ainsi une HTIC d´origine gazeuse. par ailleurs le contrôle des facteurs d´agression cérébrale secondaire d´origine systémique doit être rigoureux. La pneumocéphalie constitue une complication banale et sérieuse et fait partie des facteurs de mauvais pronostic postopératoire de la chirurgie des tumeurs de la fosse cérébrale postérieure [8].

## Conclusion

La pneumocéphalie est une complication postopératoire fréquente et banale spontanement resorbable, mais ce phénomène devient sérieux quand il est compressif realisant ainsi le signe de «Mont Fuji», respensable d´une détérioration neurologique, tel que rencontré dans le post-opératoire de l´épendymome du troisième ventricule. La pneumocéphalie compressive doit être considéré alors une urgence neurochirurgicale.
